# Resignation

**Published:** 1856-05

**Authors:** 


*Resignation.*—Dr. B. H. Lemon has resigned the post of House Physician in the Buffalo Hospitan of the Sisters of Charity, to commence practice in this city. During his six months residence, Dr. Lemon has acquitted himself with honor, and we regret the circumstances which compelled his resignation.

